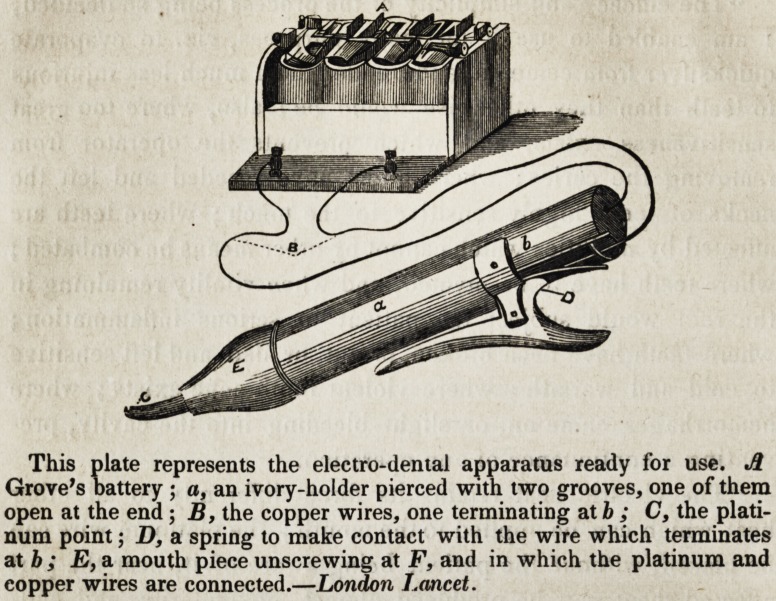# An Instrument for Applying Electric Heat in Dental Operations

**Published:** 1851-10

**Authors:** George Waite

**Affiliations:** Surgeon-Dentist.


### ARTICLE XIII.

*An Instrument for Applying Electric Heat in Dental Operations.* By George Waite, M. R. C. S., Surgeon-Dentist.

Hydrocyanic acid, as also the acetate of morphia, and strong acids, have for many years been used in dental surgery to deaden pain in teeth, but all these applications have been open to many objections.

"A conversation with the late Mr. Murphy of King's College, Cambridge, (says Mr. Waite,) suggested to me the use of electricity in dental surgery; his words, as near as I can remember, were as follows:—'The day will come when electric heat will be used in surgery, and also for many purposes in domestic arrangements.'

"The idea remained a secret with me till last year, when I communicated it to Mr. Redwood, and also to some other scientific friends, and they all appeared pleased with its simplicity and novelty.

"Early in the autumn, Mr. Laxton of Fludyer-street, entered a caveat for me at the patent office, to prevent other parties patenting the invention, although I had no idea of doing so. Considering then my plan no longer a secret, I communicated it to many friends, who expressed themselves so much pleased with it, that having obtained space at the Exposition, I forwarded a drawing of it to the illustrated catalogue of the Great Exhibition. But not to intrude too much on your valuable space, I will state, that for the purpose I use a Grove's battery with eight cells. When using it, I have in my hand a holder with two copper wires passing through it, one positive from the battery, and the other terminating in a groove in the holder and fastened to a spring, by which I make or break contact at will with the negative wire. To the further end of the two wires a thin platinum wire is connected, and on the battery being charged and contact made, this takes suddenly the electric heat.

"The efficacy and simplicity of the process being so decided, I am enabled to use it for many purposes, viz. to evaporate quicksilver from cements, and render them much less injurious to teeth than they otherwise would be; also, where too great sensitiveness exists, and which prevents the operator from removing the caries; where gums have receded and left the necks of teeth highly sensitive to the touch; where teeth are affected by mollities which cannot by other means be combated; where teeth have to be pivoted, and when vitality remaining in the root would subject the patient to serious inflammation; where teeth have been broken, or cut, or filed, and left sensitive to cold and warmth; where violent tooth-ache exists; where hemorrhages come on, or slight bleeding into the cavity, preventing a continuance of any operation.

"The electric heat retains its force differently to all other heat which can be applied to the mouth; the platinum wire can be placed, without the patient being aware of it, near the part affected, heat can be produced almost momentarily, and suddenly deadened, and as a most interesting phenomenon, and one which has surprised me very much, in patients of a highly nervous temperament where I have expected much suffering, none has been endured on its application. It would be superfluous here to detail many interesting facts which the use of electric heat will discover to the scientific dentist; these, philosophy explains with the laws of the sensitive faculty. It is my sincere hope that operators will be judicious in the use of this agent, and not bring it into disrepute by ill-judged and ill-timed applications.

"In many cases it will be found equally efficacious when holding it near the teeth, as if they were touched by it.

"Care must be taken not to continue its application too long, as it will burn up and blacken the part it touches.

"As time passes on, I look forward to its use being generally understood, and it will then give rise to many improvements tending to the benefit of society."

This plate represents the electro-dental apparatus ready for use. *A* Grove's battery; *a,* an ivory-holder pierced with two grooves, one of them open at the end; *B,* the copper wires, one terminating at *b;* *C,* the platinum point; *D,* a spring to make contact with the wire which terminates at *b;* *E,* a mouth piece unscrewing at *F,* and in which the platinum and copper wires are connected.—*London Lancet.*

## Figures and Tables

**Figure f1:**